# Mineral Phase Reconstruction and Separation Behavior of Zinc and Iron from Zinc-Containing Dust

**DOI:** 10.3390/ma16093481

**Published:** 2023-04-30

**Authors:** Zeqiang Xie, Guang Li, Yufeng Guo, Shuai Wang, Feng Chen, Lingzhi Yang, Ganghua Fu, Tao Jiang

**Affiliations:** School of Minerals Processing and Bioengineering, Central South University, Changsha 410083, Chinayfguo@csu.edu.cn (Y.G.);

**Keywords:** zinc ferrite, calcium roasting, microstructure, zinc oxide, ammonia leaching

## Abstract

Zinc-containing dust can be found in ironmaking and steelmaking, and it is an important secondary resource of zinc. Zinc-containing dust from an electric furnace was used as a raw material to study the phase transformation behavior of the dust using a calcification roasting process and the zinc–iron separation behavior by using ammonia leaching. The zinc-bearing dust was mixed with CaO and roasted to transform the zinc ferrite into zinc oxide. The results showed that increasing the calcium oxide to dust ratio could promote the conversion of zinc ferrite to zinc oxide. When the calcium oxide ratio reached 60%, the peak of zinc ferrite in the calcined-roasted product in the zinc-containing dust basically disappeared. As the temperature increased, the zinc oxide grains increased but were still smaller than 10 µm. The calcined-roasted product was crushed and ground, and the zinc was leached by ammonia. A zinc–iron recovery rate of 86.12% was achieved by the ammonia leaching. The leachate could be used for zinc extraction by electrolysis. The leaching residue was mainly calcium ferrate, which could be used in sintering production. The proposed process may achieve on-site recovery of zinc-containing dust in steel-making plants.

## 1. Introduction

Zinc can be used to coat steel materials to prevent corrosion, and it can also be alloyed with different metals [1,2]. In China, roughly 100 million tons of dust is generated each year in steelmaking plants, and the utilization rate is less than 20% [3]. China currently produces a large number of zinc-containing secondary resources; the zinc-containing phase in the majority of the dust is the zinc ferrite phase [4]. Zinc ferrite has a spinel structure with stable physicochemical properties [1], making it difficult to apply common hydrometallurgical methods and routes for recycling [5]. The recovery rate of zinc from zinc-ferrite-type dust is low, and large amounts of slag also cause environmental pollution.

The current treatment of electric-furnace dust in steel companies is based on recycling, including hydro- and pyrometallurgical treatment methods [3,6,7,8,9,10,11,12,13,14,15,16,17]. The direct recycling of zinc-containing dust in steel companies is achieved using electric-furnace dust as a raw material for the sintering processes [18,19,20]. The dust usually has a fine particle size; therefore, the permeability of the sintering layer is affected. Blast-furnace smelting limits the zinc content of the charge to a certain extent; therefore, the zinc content of the sintered or pellet-treated zinc-containing dust must not be too high [3,21]. One of the common methods of treating secondary resources is acid leaching [22,23,24]. In the acid-leaching process, the soluble zinc contained in the electric-furnace dust is dissolved in acid and incorporated into the leaching solution, after which the leaching solution is purified and then treated by electrowinning to obtain metallic zinc ingots. This enables the comprehensive recycling of zinc resources [25,26]. Zinc ferrite is difficult to dissolve; normally, a leaching rate of only 80% can be achieved at room temperature and pressure [27,28]. Alkali leaching mainly involves the application of alkaline solutions such as sodium hydroxide in the leaching process of electric-furnace dust, by which valuable metal elements are obtained [29,30]. However, the recovery rate of this method is not high.

The processes for the treatment of zinc-containing dust by direct reduction include rotary hearth furnaces, rotary kilns, and circulating fluidized bed processes. The melt reduction method can obtain a higher quality of iron and a higher dezincification rate; however, the method has drawbacks such as high energy consumption and high treatment costs [8]. Many experts around the world are beginning to study the roasting conversion of zinc ferrite, followed by the recycling of zinc and iron resources [31,32,33]. Researchers have treated zinc-containing electric-furnace dust using the magnetization roasting–magnetic separation method [21,34]. To improve the efficiency of the utilization of zinc resources, it is essential to focus on the development of technologies for the recovery of zinc-containing dust. Many investigations have reported that zinc ferrite could be decomposed by high-temperature roasting with the addition of lime [29,35,36,37]. The recovery of zinc from synthetic zinc ferrite by calcium roasting and ammonia leaching was reported in our previous paper [38]. The raw material in our previous paper [38] was synthesized zinc ferrite (ZnFe_2_O_4_) without other elements and oxides. Zinc-containing dust from steel companies generally contains CaO, SiO_2_, MgO, Al_2_O_3_, and other oxides. The phase transformation of zinc ferrite in dust in the process of calcium roasting can be affected by other components; thus, the reaction mechanism investigated here may be different from that of the synthesized zinc ferrite in our previous paper. The mechanism of the recovery of zinc from actual EAF dust by this method was not clear. Thus, it is essential to investigate the phase transformation and extraction of zinc from zinc-containing dust by the calcium roasting–ammonia-leaching process.

In this study, the effects of the addition of Ca(OH)_2_ as well as the roasting temperature and time on the phase transformation of EAF dust were investigated through XRD and SEM–EDS techniques. The optimal parameters of the ammonia-leaching method for the recovery of zinc from the calcium-roasted dust were also studied. With this study, we provide a technical basis for the highly efficient utilization of zinc-ferrite-containing dust.

## 2. Materials and Methods

### 2.1. Materials

EAF dust was obtained from a steelmaking plant in China. The XRF analysis results of the EAF dust are shown in Table 1. The EAF dust contained a high iron content of 50.28% and a zinc content of 7.5%, in addition to small amounts of calcium, manganese, sodium, chlorine, and silicon. The total iron content, SiO_2_ and ZnO content are also determined by chemical analysis and listed in Table 2. 

**Figure 1:**
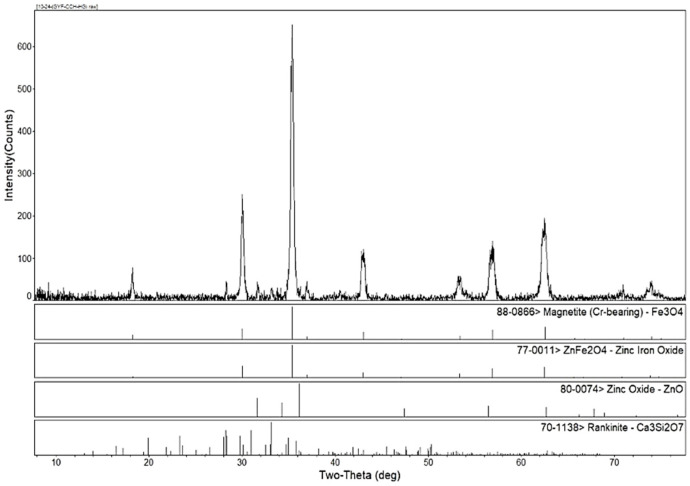
XRD pattern of EAF dust.

Figure 1 shows the XRD pattern of the EAF dust. The main phases of the EAF dust were magnetite and zinc ferrite, as well as small amounts of zinc oxide and silicate.

### 2.2. Experimental Methods

The EAF dust powder and analytical-grade CaO powder were mixed. The mixture was pressed into cylindrical pellets, and then loaded into a corundum boat and roasted in a horizontal tube furnace at roasting temperatures. The selection of the roasting temperature and CaO additions was referred to in our previous paper [38]. We studied temperatures of 1150 °C, 1200 °C, 1250 °C, and 1300 °C. The CaO addition ratios were 40%, 50%, and 60% with respect to the dust. The calcium roasting of the zinc ferrite samples was conducted in a tube furnace, which was heated by silicon–carbon rods and equipped with an automatic temperature control system. After cooling to room temperature, the samples were removed, and the roasted samples were ground to less than 200 mesh for the detection of the Zn and Fe contents as well as the analyses of the phase and microstructure.

The leaching method for the samples was also described In our previous paper. The leaching experiments were conducted in a 250 mL beaker heated in a water bath to a target temperature. The leaching procedure was initiated by pouring 100 mL of the required concentration of the ammonia-leaching agent into a 250 mL beaker. The leaching agent was then heated to the experimental temperature with stirring. The time was noted when the dust samples or calcified roasting samples were added to the leaching beaker. When the required reaction time had elapsed, the slurry was filtered. An appropriate dosage of the dilution and leaching residue was then obtained for the analysis. Each set of analyses was repeated three times and the average value was taken for the final data.

### 2.3. Analytical Methods

The zinc content of the samples was determined by EDTA complexometric titration (GB/T 8151.1-2012) [39]. The chemical composition of the dust was also determined by XRF (BRUKER, Karlsruhe, Germany, S8 TIGER). The XRF data are semiquantitative. The calibration was performed with SPECTRAplus software operated from an external PC connected to the S8 TIGER. The phase compositions of the samples were analyzed by X-ray diffraction (BRUKER, Karlsruhe, Germany, D8 Advance) with a graphite monochromator and Cu-Kα radiation in the range of 10–70° at a scanning rate of 10°/min. The Jade 6.5 software was used to analyze the phase composition of the samples. The microstructure and phase compositions of the samples were also determined by SEM (scanning electron microscope) (TESCAN, MIRA3-LMH, Brno, Czech Republic) with an EDS (energy-dispersive spectrometer) (Oxford X-MAX20, Oxford Instruments Inc., Oxford, UK). The analytical uncertainty was 5% for elements with contents higher than 20%, 10% for those with 3–20%, 20% for those with 1–3%, and 50% for those with 1% or less. The samples for the SEM–EDS analysis were coated with carbon to ensure electroconductivity.

## 3. Results and Discussion

### 3.1. Phase Composition of the Calcium Roasting of EAF Dust

**Figure 2:**
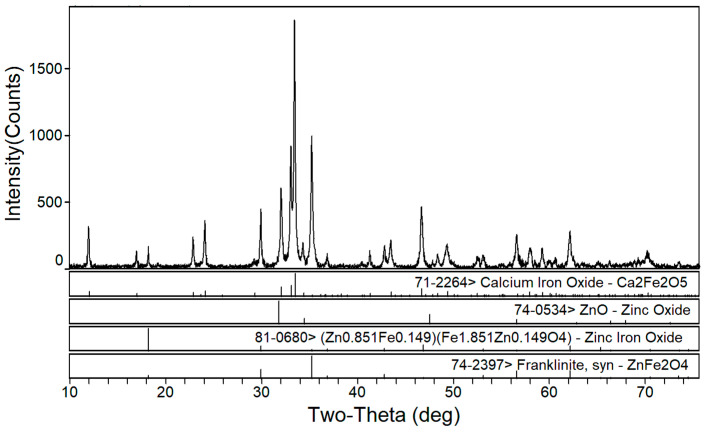
XRD patterns of EAF dust after calcination roasting with 40% calcium oxide addition.

**Figure 3:**
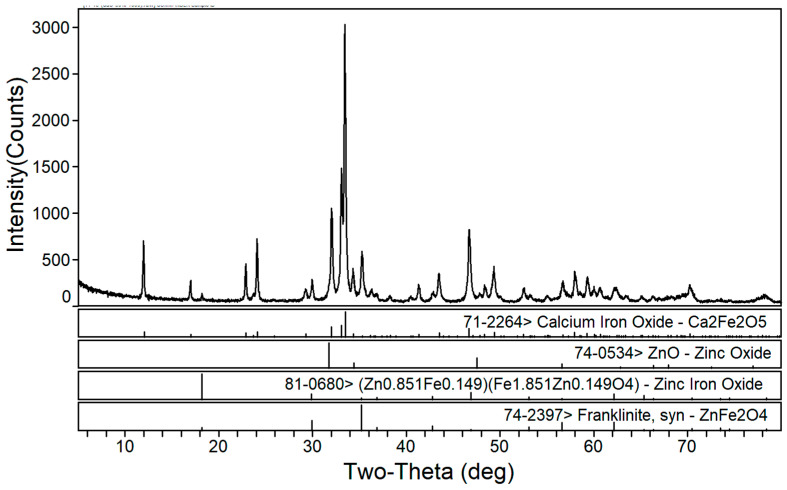
XRD pattern of EAF dust after calcination roasting with 50% calcium oxide addition.

**Figure 4:**
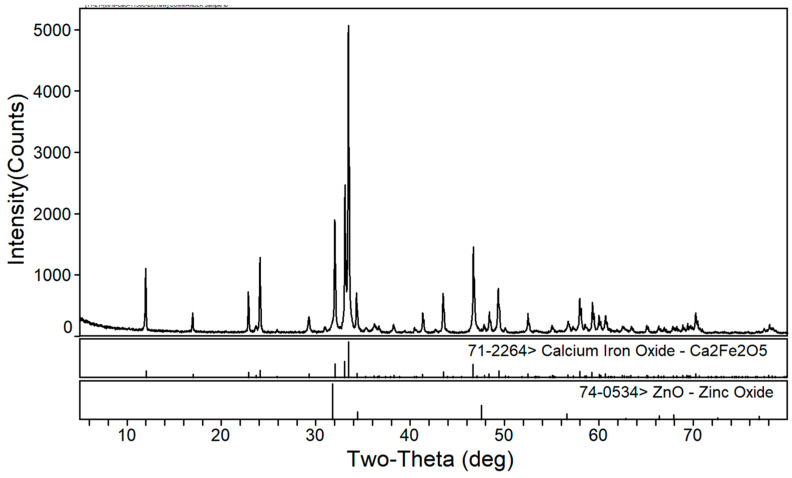
XRD pattern of EAF dust after calcination roasting with 60% calcium oxide addition.

The phase transformation of zinc ferrite by calcium roasting was reported in our previous paper [38]. The effect of the calcium oxide to dust ratio on the calcination product phase composition of the EAF dust was studied under a roasting temperature of 1150 °C and a roasting time of 2 h. Figure 2, Figure 3 and Figure 4 are the XRD patterns of the roasted samples with 40%, 50%, and 60% calcium oxide ratios, respectively. Zinc ferrite and dicalcium ferrite were the main phases of the calcination products in the zinc-containing dust with different proportions of calcium oxide.

Figure 2 shows the XRD pattern of the calcined products in the zinc-containing dust with a 40% calcium oxide ratio. The main phases of the roasting product at a 40% ratio were dicalcium ferrite (Ca_2_Fe_2_O_5_), residual unreacted zinc ferrite (ZnFe_2_O_4_), and a small amount of newly generated zinc oxide. According to thermodynamic analyses [38], under standard conditions, the ΔG of the formation reaction of calcium oxide and ferric oxide to calcium ferrite is much lower than the ΔG of the reaction of calcium oxide and zinc ferrite to calcium ferrite and zinc oxide, indicating that the added calcium oxide first reacts with iron oxide to form dicalcium ferrite. The obvious zinc ferrite peaks in the XRD indicated that 40% calcium oxide was insufficient to achieve a complete reaction of the zinc ferrite.

Figure 3 shows the XRD pattern of the calcined products of the EAF dust with 50% calcium oxide. Compared with Figure 2, it can be seen that with the increase in calcium oxide, the diffraction peaks of dicalcium ferrite were significantly enhanced whereas those of zinc ferrite significantly decreased. As shown in Figure 4, when the dosage of calcium oxide increased to 60%, there were no diffraction peaks of zinc ferrite in the phase of the roasted product and the diffraction peaks of zinc oxide were more obvious, which indicated that the zinc ferrite was almost completely transformed under the condition of 60% calcium oxide.

**Figure 5:**
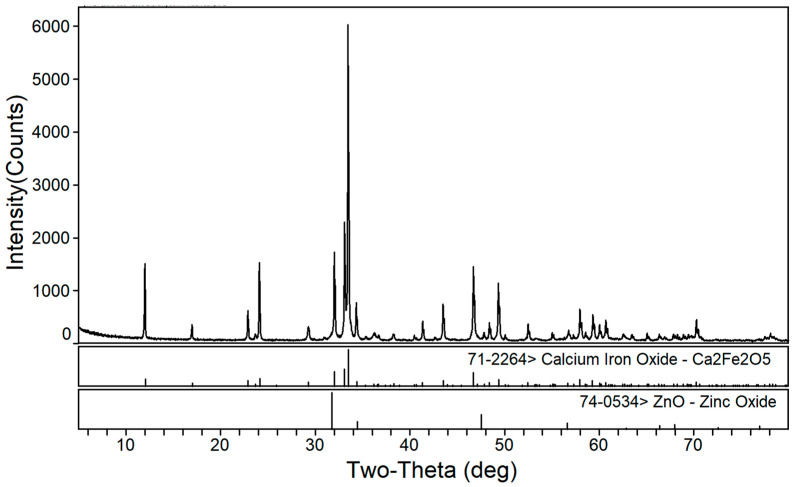
XRD patterns of roasted EAF dust (addition of 60% calcium oxide at 1200 °C for 2 h).

**Figure 6:**
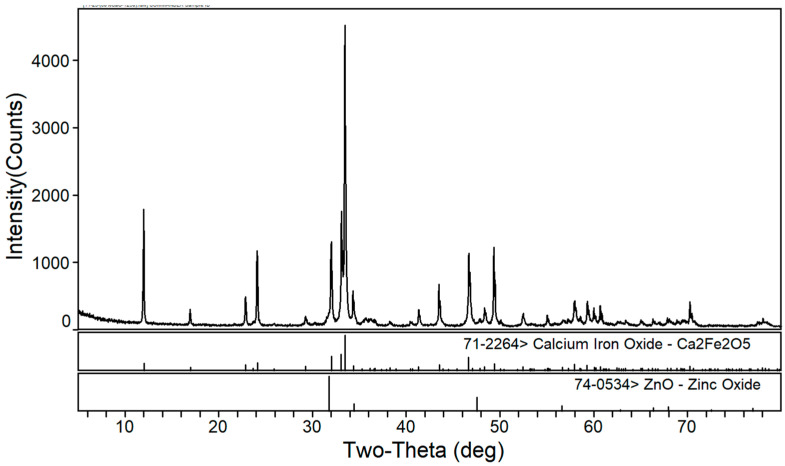
XRD patterns of roasted EAF dust (addition of 60% calcium oxide at 1250 °C for 2 h).

Figure 5 and Figure 6 show the XRD patterns of the roasted EAF dust with a fixed calcium oxide ratio of 60%, a roasting time of 2 h, and roasting temperatures of 1200 °C and 1250 °C, respectively. With the increase in roasting temperature, the phase composition of the roasted products in the zinc-containing dust (calcium ferrite and zinc oxide) remained unchanged. In addition, the roasted sample appeared to be partly melted at 1250 °C; therefore, when the temperature increased to 1300 °C, complete melting of the roasted sample occurred. Thus, the roasting temperature should not exceed 1250 °C.

**Figure 7:**
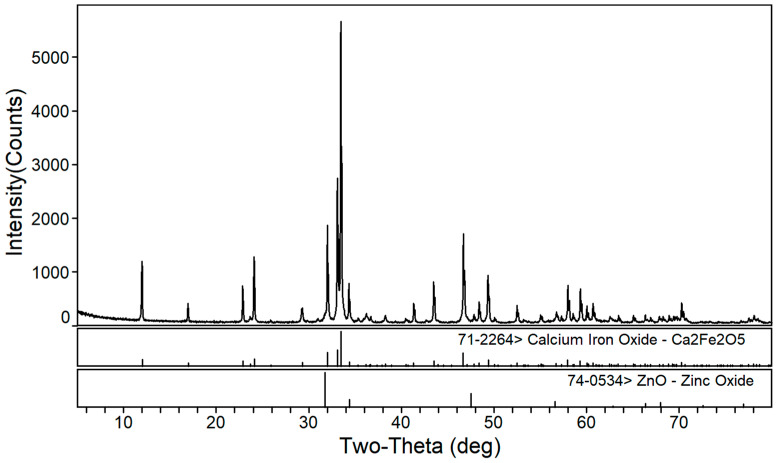
XRD patterns of roasted EAF dust (addition of 60% calcium oxide at 1250 °C for 3 h).

Figure 7 shows the XRD pattern of the calcined products of the EAF dust at a roasting temperature of 1250 °C and a roasting time of 3 h with a 60% calcium oxide ratio. By comparing Figure 6 and Figure 7, it can be seen that with an extension of the roasting time, the change in the phase composition of the roasted products was insignificant.

### 3.2. Microstructure of the Calcined Products of EAF Dust

The phase analysis of the calcination product showed that the zinc ferrite in the calcination product was essentially converted to zinc oxide when the proportion of calcium oxide reached 60%. SEM–EDS was used to analyze the microstructure of the roasted products at different roasting temperatures and times under the condition of a 60% calcium oxide ratio.

**Figure 8:**
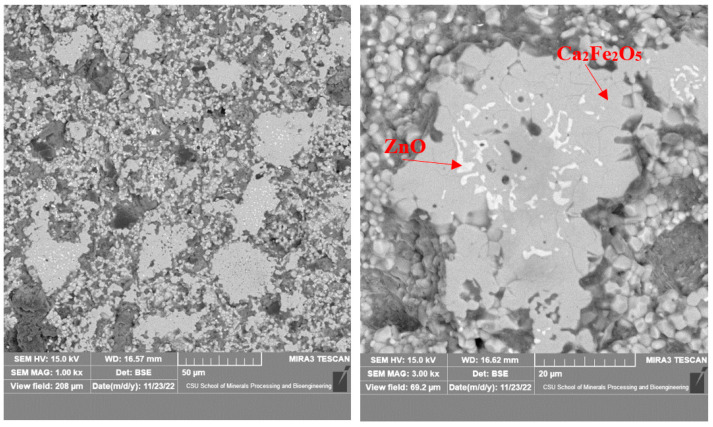
Microstructure of calcined products of EAF dust (1150 °C; 2 h).

**Figure 9:**
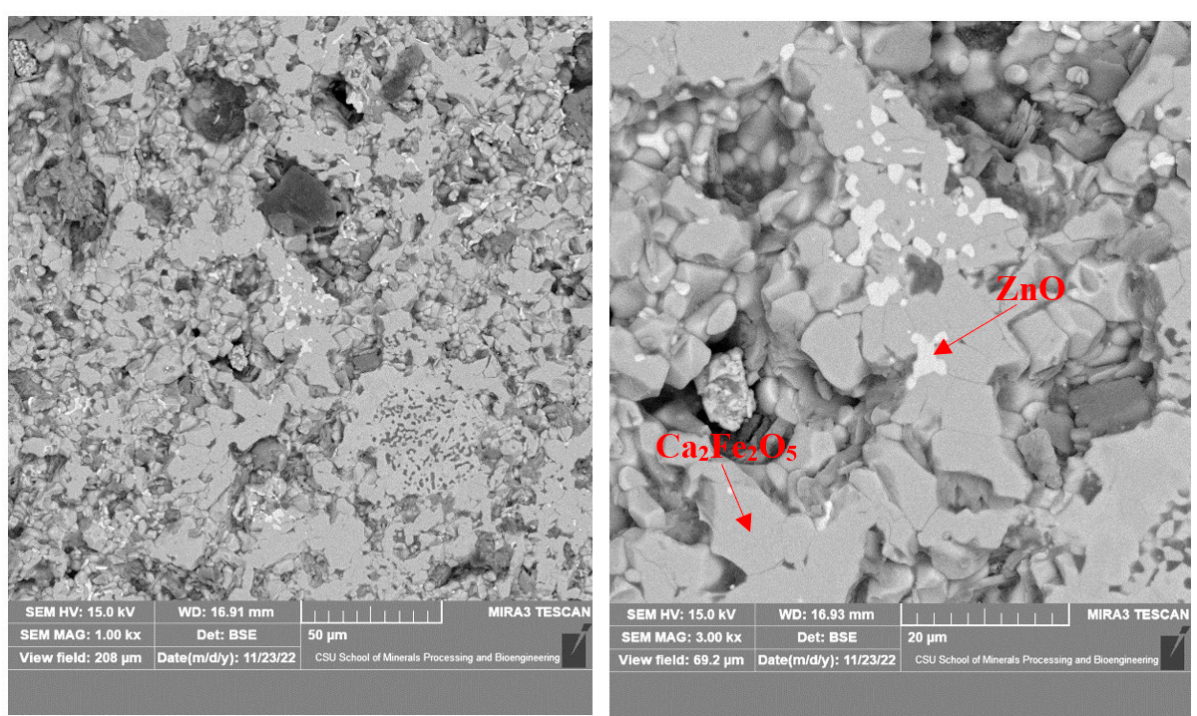
Microstructure of calcined products of EAF dust (1200 °C; 2 h).

As shown in Figure 8, there were mainly two gray phases in the products roasted at 1150 °C, in which the gray was zinc oxide and the dark gray was calcium ferrite. The zinc oxide grains were small; about 5 μm, at most. They were mainly coated with calcium ferrite and had a complex distribution that was mainly located in the middle of the calcium ferrite matrix. Figure 9 shows that with the increase in temperature, the grain size of ZnO increased, but it remained lower than 10 μm. When the calcination temperature reached 1250 °C, the newly formed zinc oxide precipitated at the grain boundary of the calcium ferrite, with a small grain size and an irregular shape.

**Figure 10:**
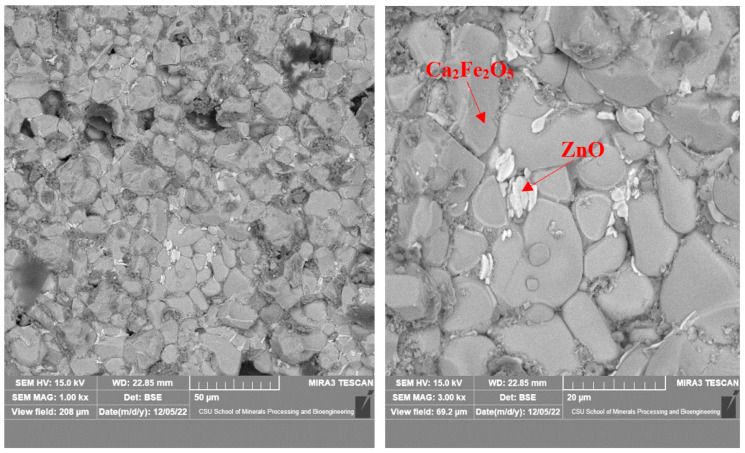
Microstructure of calcined products of EAF dust (1250 °C; 2 h).

**Figure 11:**
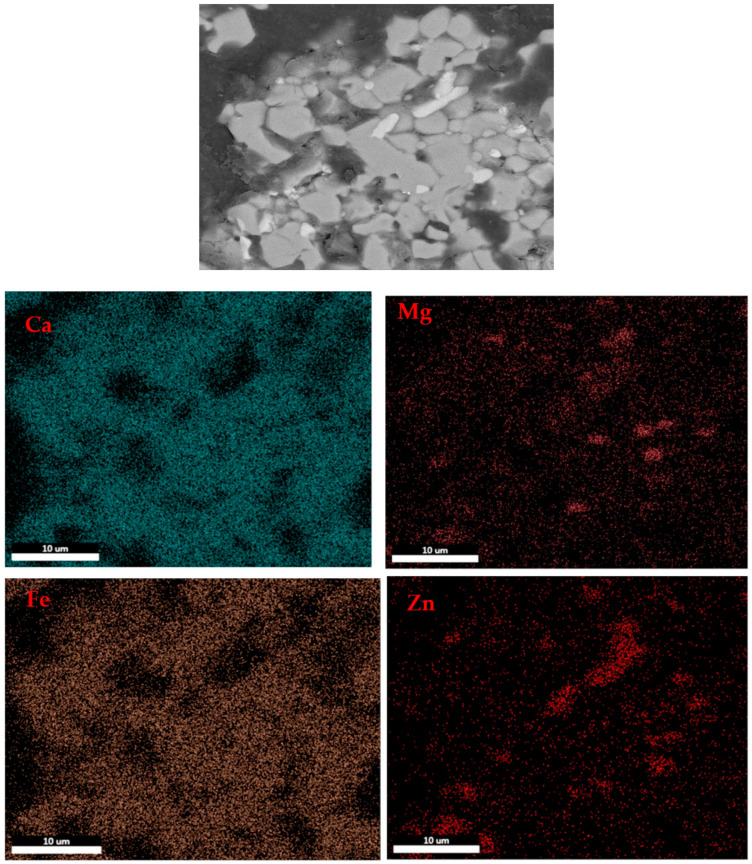
Elemental distribution of the roasted products at 1250 °C.

As shown in Figure 10 and Figure 11, when the roasting temperature rose to 1250 °C, the grains of zinc ferrite transformed into zinc oxide after the roasting of the zinc dust, with a certain degree of aggregation. Due to the low zinc content in the zinc dust, the aggregate growth of the zinc oxide was also limited. Under the condition of 1250 °C, the zinc ferrite in the dust containing zinc could be converted into zinc oxide, which provided the basis for the subsequent recovery of the zinc. Figure 11 shows the distribution of the elements in the products after roasting. It was clear that the zinc and iron were distributed in different phases.

**Figure 12:**
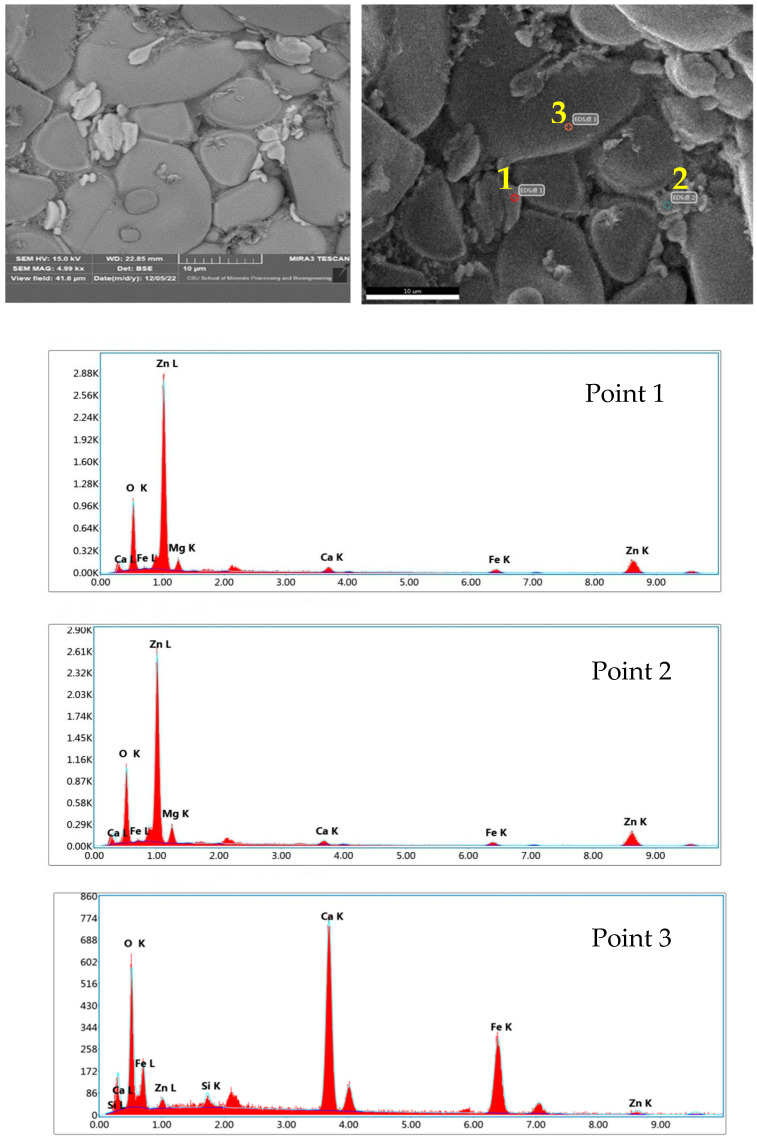
EDS analysis of calcination products of EAF dust.

Figure 12 and Table 3 show the EDS analysis of the products after roasting at 1250 °C for 2 h. Small amounts of iron, calcium, and magnesium remained in the zinc oxide phase whereas the calcium ferrite contained a small amount of zinc, presumably as a solid solution [40].

**Figure 13:**
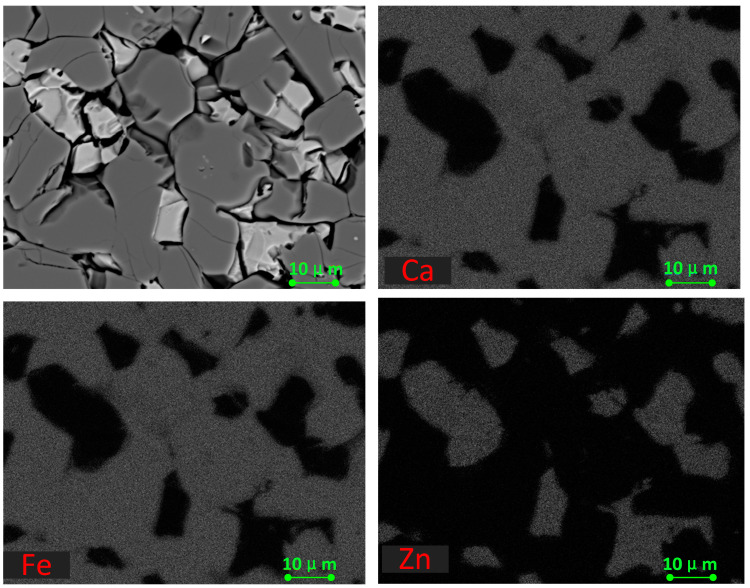
Main element distribution of the roasted pure zinc ferrite with the roasting time of 2 h at 1220 °C [38].

Figure 13 illustrates the elemental mapping of the roasted pure zinc ferrite at 1220 °C for 2 h, as discussed in our previous paper [38]. It can be seen that the zinc element migrated to and gathered in the bright white area (ZnO phase zones) and the Ca and Fe elements migrated to and accumulated in the dark grey area (Ca_2_Fe_2_O_5_ phase accumulation area). Compared with Figure 11, the size of the ZnO grains in the dust was smaller than that in the pure zinc ferrite, probably due to the existence of other oxides such as Fe_2_O_3_ and MgO, SiO_2_, which prevent the migration of zinc during the roasting process. Furthermore, according to our previous investigation [38], the addition of lime in the actual dust is higher than that for the pure zinc ferrite due to the other oxides that would react with lime in the roasting process.

### 3.3. Separation of Iron and Zinc from the Calcined Products of EAF Dust by Ammonia Leaching

**Figure 14:**
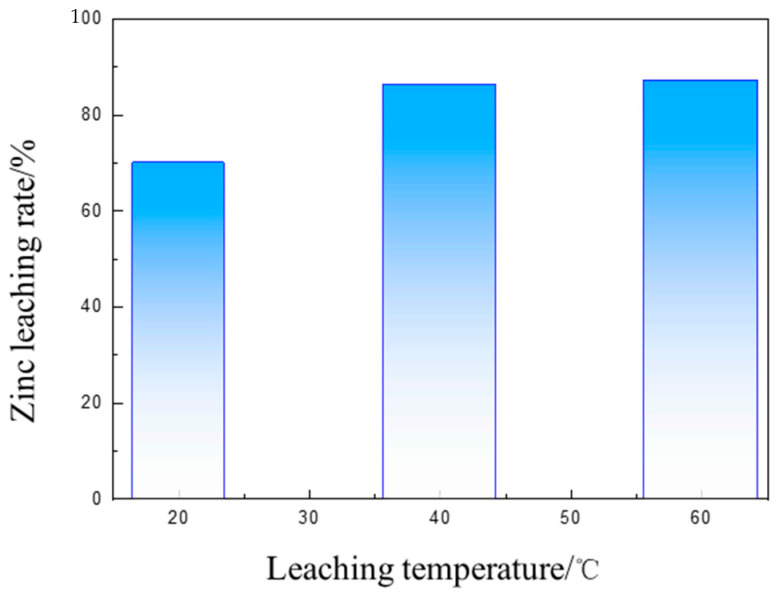
The impact of leaching temperature on the zinc leaching rate of roasted dust.

Ammonia-leaching experiments were conducted on the samples obtained by roasting at 1250 °C for 2 h with a 60% addition of calcium oxide. The ammonia-leaching solution was prepared by mixing NH_4_Cl, NH_3_·H_2_O, and H_2_O. The total ammonia concentration was 6 mol/L, the mole ratio (NH_4_Cl)/(NH_3_·H_2_O) was 1, the solid–liquid ratio was 100 g/L, the time was 3 h, and the stirring rate was 300 r/min. The effect of the leaching temperature on the zinc leaching rate was studied under the above conditions. As shown in Figure 14, the leaching rate correspondingly increased with the gradual increase in temperature. There was only a small increase in the leaching rate with temperatures from 40 to 50 °C. As higher temperatures favor the volatilization of ammonia, we suggest that 40 °C is an appropriate temperature for the leaching process.

**Figure 15:**
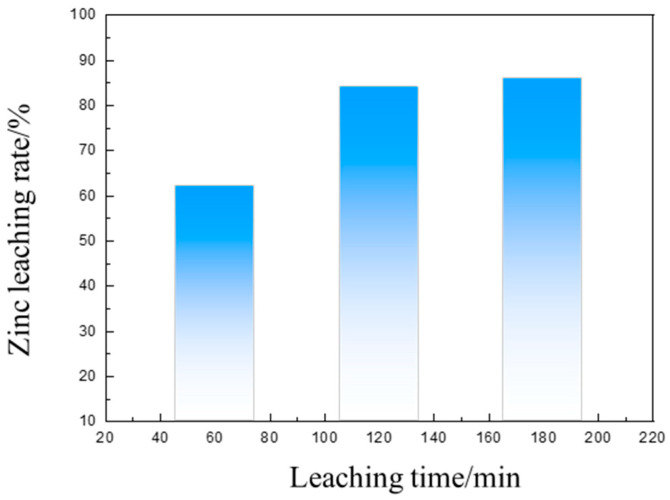
The impact of leaching time on the zinc leaching rate of roasted dust.

The effect of the leaching time on the ammonia-leaching rate of the calcined products in the zinc dust was studied under the conditions of a leaching temperature of 40 °C, a solid–liquid ratio of 100 g/L, a stirring rate of 300 r/min, a total ammonia concentration of 6 mol/L, and n (NH_4_Cl)/n (NH_3_·H_2_O) = 1. Figure 15 shows the effect of the leaching time on the zinc leaching rate.

In Figure 15, it can clearly be seen that the zinc leaching rate sharply increased at first, and then there were only minor changes with the extension of time. When the leaching time was less than 100 min, the leaching rate significantly increased with the increase in time. After the leaching time exceeded 120 min, there were only minor changes to the zinc leaching rate with the leaching time, and the leaching reaction was predominantly completed after 2 h.

**Figure 16:**
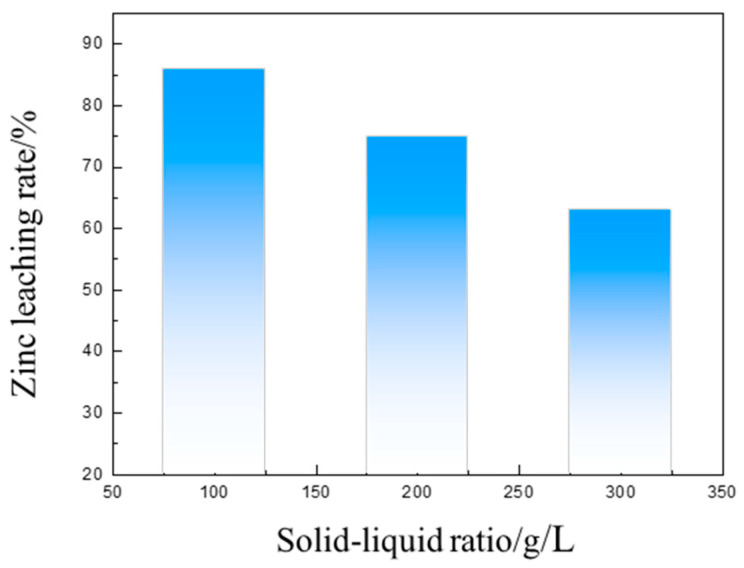
The influence of the solid to liquid ratio on the zinc leaching rate.

The influence of the solid–liquid ratio on the zinc leaching rate under the condition of n (NH_4_Cl)/n (NH_3_·H_2_O) = 1, a leaching temperature of 40 °C, a leaching time of 3 h, a stirring rate of 300 r/min, and a total ammonia concentration of 6 mol/L was then investigated. Figure 16 shows that the leaching rate decreased with an increase in the solid–liquid ratio. The optimal solid–liquid ratio was 100 g/L.

**Figure 17:**
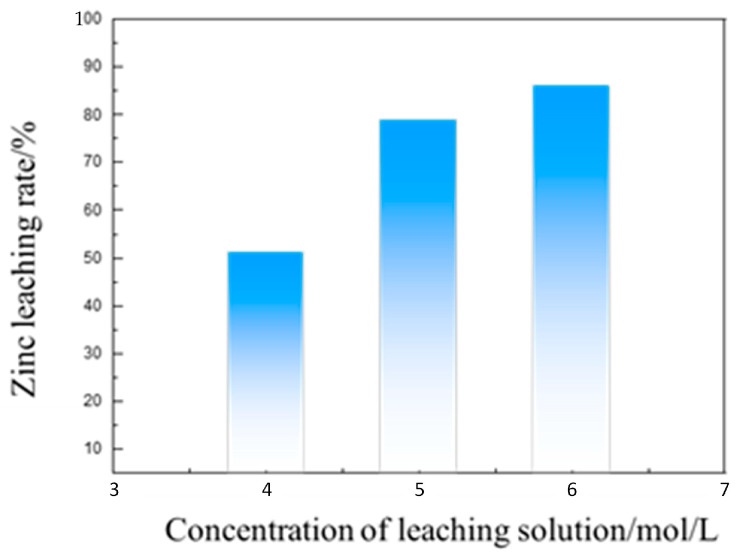
The impact of the concentration of the leaching solution on the zinc leaching rate.

Figure 17 shows the effect of the leaching solution concentration on the zinc leaching rate. Due to the dissolution of zinc in ammonia, leaching systems consume a certain amount of reagents. Too low a concentration can lead to incomplete reactions; thus, the concentration of the leaching solution should be greater than 6 mol/L to ensure the adequate leaching of zinc.

### 3.4. Proposed Process for the Comprehensive Utilization of Zinc and Iron in EAF Dust

**Figure 18:**
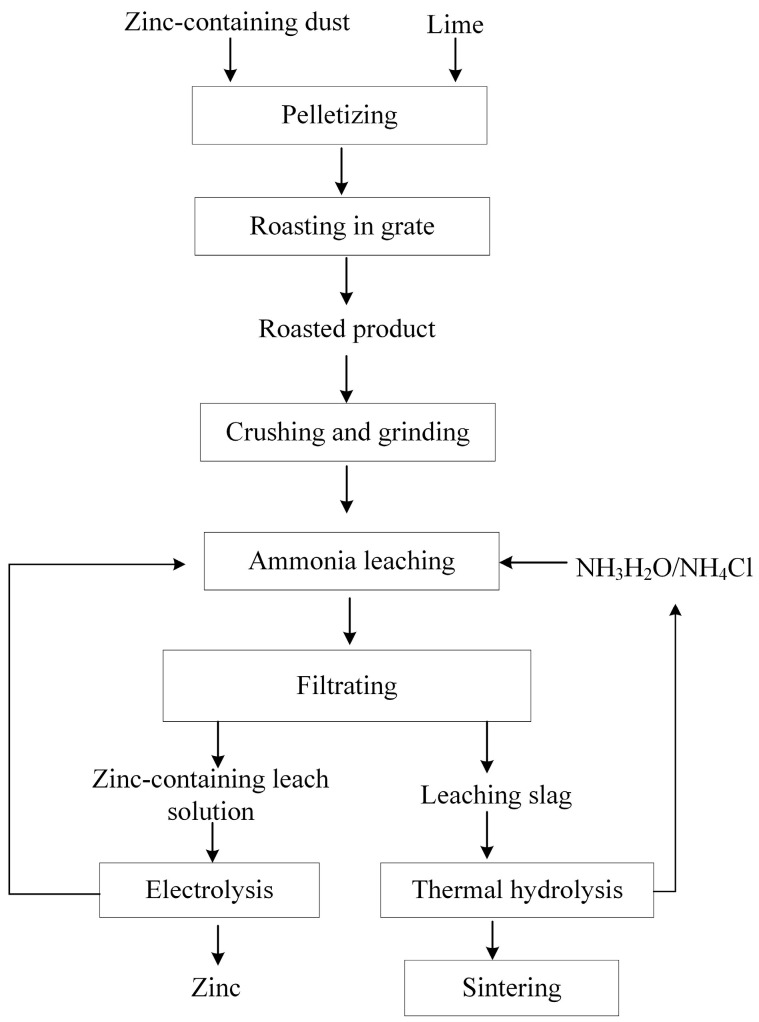
Flowchart of zinc–iron separation and the utilization process for zinc-containing dust.

According to the above results, ammonia leaching was more suitable for the separation process of zinc and iron following an ore phase reconstruction by the roasting of zinc dust. As shown in Figure 18, the zinc-containing dust was mixed with a calcium-containing conversion agent and converted into blocks before calcium roasting. The products after calcium roasting were crushed and ground; subsequently, the zinc was removed using the ammonia-leaching method. The leaching solution could be used for electrolytic zinc extraction and improving the recovery of zinc resources. Zinc dust could be applied in sintering production.

## 4. Conclusions

The phase transformation and the extraction of zinc from zinc-containing dust by a calcium roasting–ammonia-leaching process were investigated in this study. The transformation of zinc ferrite in the dust could be completed at 1250 °C with a 60% calcium oxide ratio. The increase in the roasting temperature was conducive to the growth of zinc oxide grains, but the zinc ferrite grains remained smaller than 10 microns, which was not optimal for the subsequent grinding separation. The roasted products were leached by the ammonia method, and the recovery rate of zinc reached 86.12% under optimum ammonia-leaching conditions.

A suitable separation process for zinc recovery from zinc ferrite dust should include the following steps: Calcium calcination should be performed after the calcium calcination products have been crushed and ground, and zinc should be extracted by ammonia leaching. The leaching solution should be electrolyzed to extract the zinc. The leaching slag would consist mainly of calcium ferrite, which can be used as prefabricated calcium ferrite for sintering production to recycle zinc-containing dust in ironmaking and steelmaking companies.

## Figures and Tables

**Table 1:** Chemical composition of zinc-containing electric-furnace dust by XRF/wt%.

Fe	O	Zn	Ca	Mn	Na	Cl	Si
50	29	8	3	2	2	2	1

**Table 2:** Main chemical composition of zinc-containing dust by chemical analysis/wt%.

Tfe	SiO_2_	ZnO
52.34	2.27	9.41

**Table 3:** EDS composition of calcination products of EAF dust.

Element	1 (ZnO)	2 (ZnO)	3 (Ca_2_Fe_2_O_5_)
O	16.11	16.83	16.57
Mg	3.98	5.96	0.95
Ca	3.06	2.48	34.35
Fe	6.07	5.45	41.68
Zn	70.79	69.28	6.45

## Data Availability

Not applicable.
